# Automatic segmentation of esophageal cancer, metastatic lymph nodes and their adjacent structures in CTA images based on the UperNet Swin network

**DOI:** 10.1002/cam4.70188

**Published:** 2024-09-20

**Authors:** Runyuan Wang, Xingcai Chen, Xiaoqin Zhang, Ping He, Jinfeng Ma, Huilin Cui, Ximei Cao, Yongjian Nian, Ximing Xu, Wei Wu, Yi Wu

**Affiliations:** ^1^ Department of Digital Medicine, College of Biomedical Engineering and Medical Imaging Army Medical University (Third Military Medical University) Chongqing China; ^2^ Department of Histology and Embryology Shanxi Medical University Taiyuan China; ^3^ Department of Cardiac Surgery, Southwest Hospital Army Medical University (Third Military Medical University) Chongqing China; ^4^ Department of General Surgery Shanxi Province Cancer Hospital/Shanxi Hospital Affiliated to Cancer Hospital, Chinese Academy of Medical Sciences/Cancer Hospital Affiliated to Shanxi Medical University Taiyuan China; ^5^ Ministry of Education Key Laboratory of Child Development and Disorders, National Clinical Research Center for Child Health and Disorders Children's Hospital of Chongqing Medical University Chongqing China; ^6^ Department of Thoracic Surgery, Southwest Hospital Army Medical University (Third Military Medical University) Chongqing China; ^7^ Yu‐Yue Pathology Research Center Jinfeng Laboratory Chongqing China

**Keywords:** 3D reconstruction, automatic segmentation, computed tomography angiography (CTA), deep learning, esophageal cancer

## Abstract

**Objective:**

To create a deep‐learning automatic segmentation model for esophageal cancer (EC), metastatic lymph nodes (MLNs) and their adjacent structures using the UperNet Swin network and computed tomography angiography (CTA) images and to improve the effectiveness and precision of EC automatic segmentation and TN stage diagnosis.

**Methods:**

Attention U‐Net, UperNet Swin, UNet++ and UNet were used to train the EC segmentation model to automatically segment the EC, esophagus, pericardium, aorta and MLN from CTA images of 182 patients with postoperative pathologically proven EC. The Dice similarity coefficient (DSC), sensitivity, and positive predictive value (PPV) were used to assess their segmentation effectiveness. The volume of EC was calculated using the segmentation results, and the outcomes and times of automatic and human segmentation were compared. All statistical analyses were completed using SPSS 25.0 software.

**Results:**

Among the four EC autosegmentation models, the UperNet Swin had the best autosegmentation results with a DSC of 0.7820 and the highest values of EC sensitivity and PPV. The esophagus, pericardium, aorta and MLN had DSCs of 0.7298, 0.9664, 0.9496 and 0.5091. The DSCs of the UperNet Swin were 0.6164, 0.7842, 0.8190, and 0.7259 for T1‐4 EC. The volume of EC and its adjacent structures between the ground truth and UperNet Swin model were not significantly different.

**Conclusions:**

The UperNet Swin showed excellent efficiency in autosegmentation and volume measurement of EC, MLN and its adjacent structures in different T stage, which can help to T and N stage diagnose EC and will save clinicians time and energy.

## Graphical abstract

The UperNet Swin model showed higher efficiency in autosegmentation and volume measurement of EC (esophageal cancer) and its adjacent structures in different T and N stages, which can help to T and N stage diagnose EC and will save clinicians time and energy.

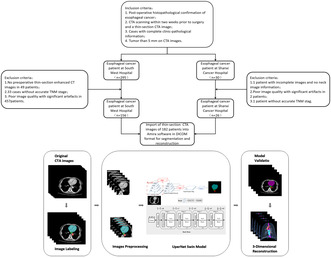

## 1 INTRODUCTION

Esophageal cancer (EC) is the seventh most common cancer and the sixth leading cause of cancer death worldwide,^1^, ^2^ with an estimated 572,000 new cases and 509,000 deaths in 2018,^2^ causing serious impacts on human health and quality of life.^3^ CT scans are crucial for the examination of EC, and accurate segmentation, 3D reconstruction, and 3D morphological quantification of EC on CT images are crucial for the accurate T and N stage diagnosis of EC, the choice of a treatment strategy, and the prognostic assessment of treatment.^4^, ^5^ Esophagoscopy is the gold standard for EC diagnosis^6^ and can confirm the diagnosis of EC. However, it is invasive and expensive, and it cannot be used to T and N stages diagnose EC, and it cannot show the 3D shape and spatial relationship between cancer and adjacent structures. EC can also metastasize in a multidirectional way via lymph nodes in the para‐esophageal, neck, abdominal cavity, and mediastinum,^7^ so for the diagnosis of EC, metastasis of lymph nodes also needs to be identified.

Multirow CT or CTA images have recently been discovered to increase the precision of EC TNM stage diagnosis.^8^, ^9^ We are committed to developing automatic segmentation models for EC to lessen the workload of doctors and increase effectiveness because manual segmentation of EC is hard and time‐consuming work and prone to segmentation errors.

Currently, tumor volume measurement is mostly used to predict T‐stage in colorectal cancer, nasopharyngeal carcinoma and non‐small cell lung cancer, which helps in accurate diagnosis and prognostic assessment, but it is less used to predict T‐stage in EC.^10^, ^11^, ^12^ In our previous study, based on CTA 3D reconstruction of EC, we can clearly observe EC's location, 3D shape and spatial relationship. We also found that EC's volume, major and minor axis are significantly predictable factors to the T‐stage diagnosis of the tumor before surgery.^13^, ^14^ In traditional acknowledge, N stage diagnosis is determined by metastatic lymph node's number.

Omeroglu S et al. showed that in patients with colorectal cancer, an MLN which size was greater than 1.05 cm may predict a poorer prognosis and lower survival in patients with stage III CRC. Maximum MLN size may be used as a surrogate for MLN number when predicting prognosis or staging patients with CRC.^15^, ^16^ So we think metastatic lymph nodes' volume is correlative with lymph nodes' number. Therefore, tumor and metastatic lymph node's segmentation and 3D calculation can determine EC'T and N stage diagnosis.

In recent years, with the rapid development of deep learning, many automatic segmentation models based on convolutional neural networks have been proposed and widely used for automatic recognition and segmentation of medical images. The U‐Net model proposed by O. Ronneberger et al.^17^ at the MICCAI conference in 2015, the UperNet proposed by Xiao T et al.^18^ in 2018, and the Swin Transformer proposed by Liu Z et al.^19^ are widely used in the field of computer vision tasks.

The twin transformer network can be used in the field of microscopic feature recognition of alloys, such as in the research of Liu P et al.^20^ and can accurately identify the microscopic features of 2.25Cr1Mo0.25 V steel for understanding the mechanism of hydrogen embrittlement (HE) and evaluating the anti‐HE performance of alloys. Nevertheless, it is rarely used in medical image segmentation. The segmentation of EC and its MLN has rarely been reported.

Jin L et al.^21^ used CT images to segment EC, and Zhang P. et al.^22^ used barium esophagrams to automatically identify EC. They only identified and segmented EC and did not accurately segment the MLN and the adjacent structures around EC, so the accurate segmentation of EC, MLN and its adjacent structures is very important.

Therefore, to improve the segmentation accuracy of EC, increase the segmentation efficiency and shorten the segmentation time, we aim to use the UperNet Swin network to create an intelligent segmentation model for EC, which can help to TN stage diagnosis and treatment decision, and help clinicians accurately diagnose EC in the TN stage, reduce clinicians' workload and improve work efficiency.

## 2 MATERIALS AND METHODS

**FIGURE 1:**
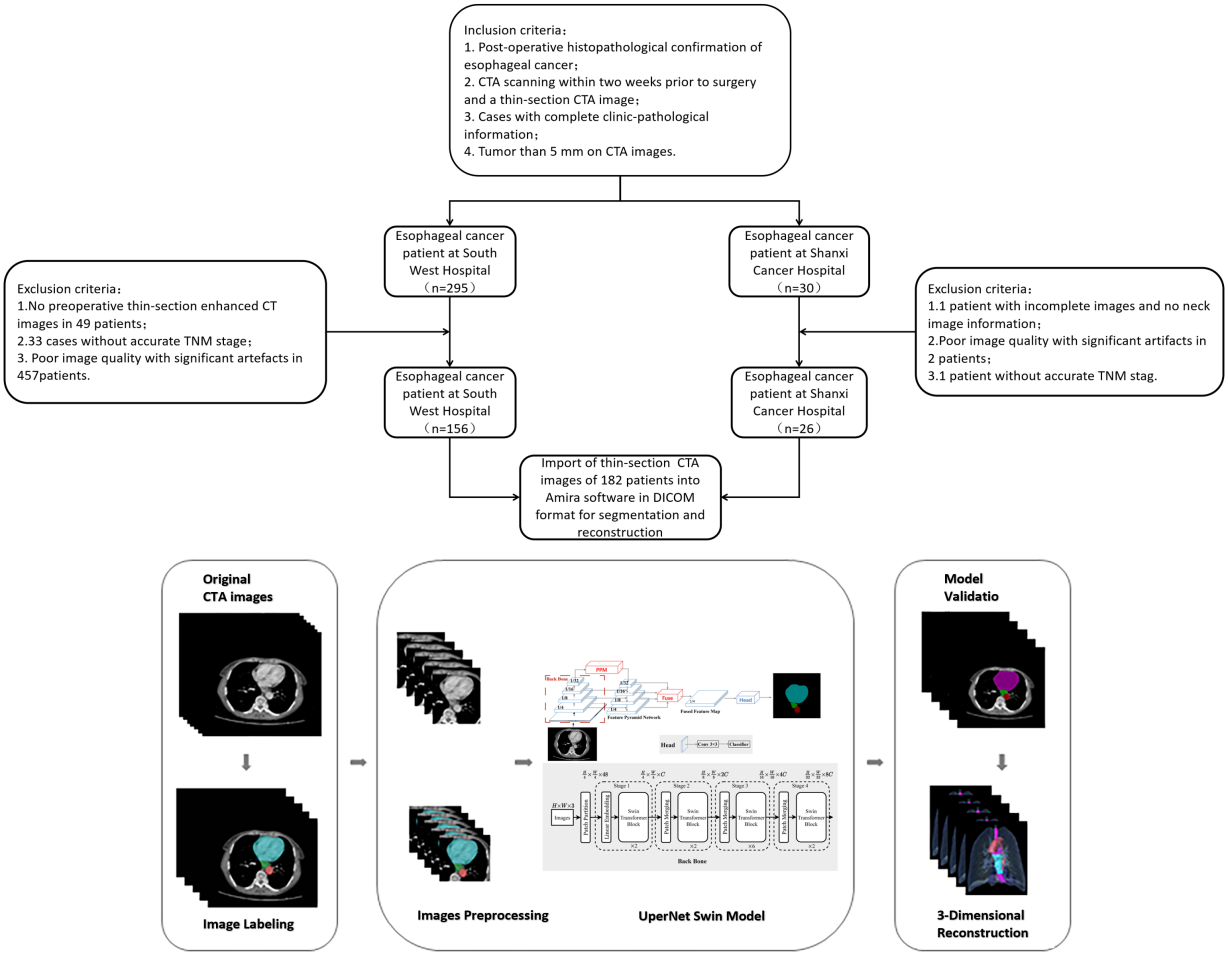
Flow chart of our research. The obtained CTA images are first manually segmented, preprocessed by cropping, windowing, rotation, etc., and then fed into the UperNet Swin network for model training to obtain a prediction map. Finally, the prediction images are used for 3D reconstruction to evaluate the model performance from both 2D and 3D aspects.

The flowchart of this experiment is shown in Figure 1.

### 2.1 Data Information

The dataset used in this experiment is a cohort of 156 patients from December 2018 to September 2022 in the First Affiliated Hospital of Army Medical University (Southwest Hospital) and 26 EC cases from April 2020 to April 2021 in Shanxi Cancer Hospital, and all images were obtained using 256 multirow CT scanners for chest CTA scanning. The study was conducted according to the Declaration of Helsinki, and approved by the Medical Ethics Committee of the First Affiliated Hospital of Army Military Medical University (No.(B)KY2021165). The data that support the findings of this study are available from the corresponding author upon reasonable request.

We enrolled a total of 182 EC patients in the study according to the American Joint Committee on Cancer (AJCC) 8th edition cancer staging guidelines^23^ after excluding some data, such as nonthin CTA images, images with poor image quality or artifacts that significantly affected the experiments, and the patients from combing EC and other malignant tumor. We confirmed the number of metastatic lymph nodes by postoperative pathology, and segmented them. A total of 17,205 preoperative CTA images were collected from the 182 patients, and the details of the case information are shown in Table 1.

**TABLE 1:** Clinical characteristics of patients in training and validation sets.

Characteristics	Training set (*n* = 142)	Validation set (*n* = 40)	*F*	*p*‐value^a^
Gender				
Male	121 (76.6%)	37 (23.4%)	1.444	0.231
Female	21 (87.5%)	3 (12.5%)		
Age, mean ± SD	61.74 ± 7.587	63.65 ± 8.288	1.924	0.167
T‐stage				
T1	22 (81.5%)	5 (18.5%)	0.443	0.507
T2	29 (78.4%)	8 (21.6%)		
T3	81 (81.0%)	19 (19.0%)		
T4	10 (55.6%)	8 (44.4%)		
N‐stage				
N0	92 (84.4%)	17 (15.6%)	3.646	0.058
N1	34 (73.9%)	12 (26.1%)		
N2	12 (57.1%)	9 (42.9%)		
N3	4 (66.7%)	2 (33.3%)		
Tumor location				
Upper	15 (68.2%)	7 (31.8%)	0.014	0.907
Median	89 (82.4%)	19 (17.6%)		
Lower	38 (73.1%)	14 (26.9%)		
Clinical stage				
I	39 (88.6%)	5 (11.4%)	3.532	0.062
II	53 (67.9%)	25 (32.1%)		
III	46 (85.2%)	8 (14.8%)		
IV	4 (66.7%)	2 (33.3%)		
Neurovascular invasion				
NO	110 (80.3%)	27 (19.7%)	1.662	0.199
YES	32 (71.1%)	13 (28.9%)		
Pathological differentiation degree				
Well differentiated	22 (75.9%)	7 (24.1%)	0.355	0.552
Moderately differentiated	93 (80.9%)	22 (19.1%)		
Poorly differentiated	27 (71.1%)	11 (28.9%)		

*Note*: Date are expressed as number (%) and mean ± SD, depending on variable distribution.

^a^ *p* < 0.05 is considered statistically significant.

### 2.2 Image segmentation and preprocessing

We imported each thin layer enhanced CT image with a thickness of 1–2 mm from the workstation in DICOM format into Amira, manually segmented the EC in the CTA image to obtain an accurate ground truth (GT), and calculated the volume size of the GT. The EC, normal esophagus, MLN, pericardium, aorta, bronchial area and lung in the CTA images were segmented by two experienced imaging physicians, and we also recorded the structures' volume and the time of manual segmentation of each structure.

For the delineation of each tumor region, the gross tumor volume (GTV) was drawn along the outline of the EC around the total tumor volume, and any pixels with attenuation less than −50 HU were excluded to avoid interference from the surrounding adjacent air, fat, blood vessels and bones on the tumor ROI delineation. When there was uncertainty about the tumor area, this region was not included in the tumor delineation.^24^, ^25^

We preprocessed the input data, including image cropping, intensity normalization, and resolution normalization. The size of the original CTA image was 512 × 512 × 420, and the range of the cancer and its surrounding structures did not exceed 320 × 320 × 16 in all the images. Therefore, we focus on the tumor area in all images, cropped the CTA images to 320 × 320 × 16 and then used them for the deep learning modeling.^21^

### 2.3 Autosegmentation Network

**FIGURE 2:**
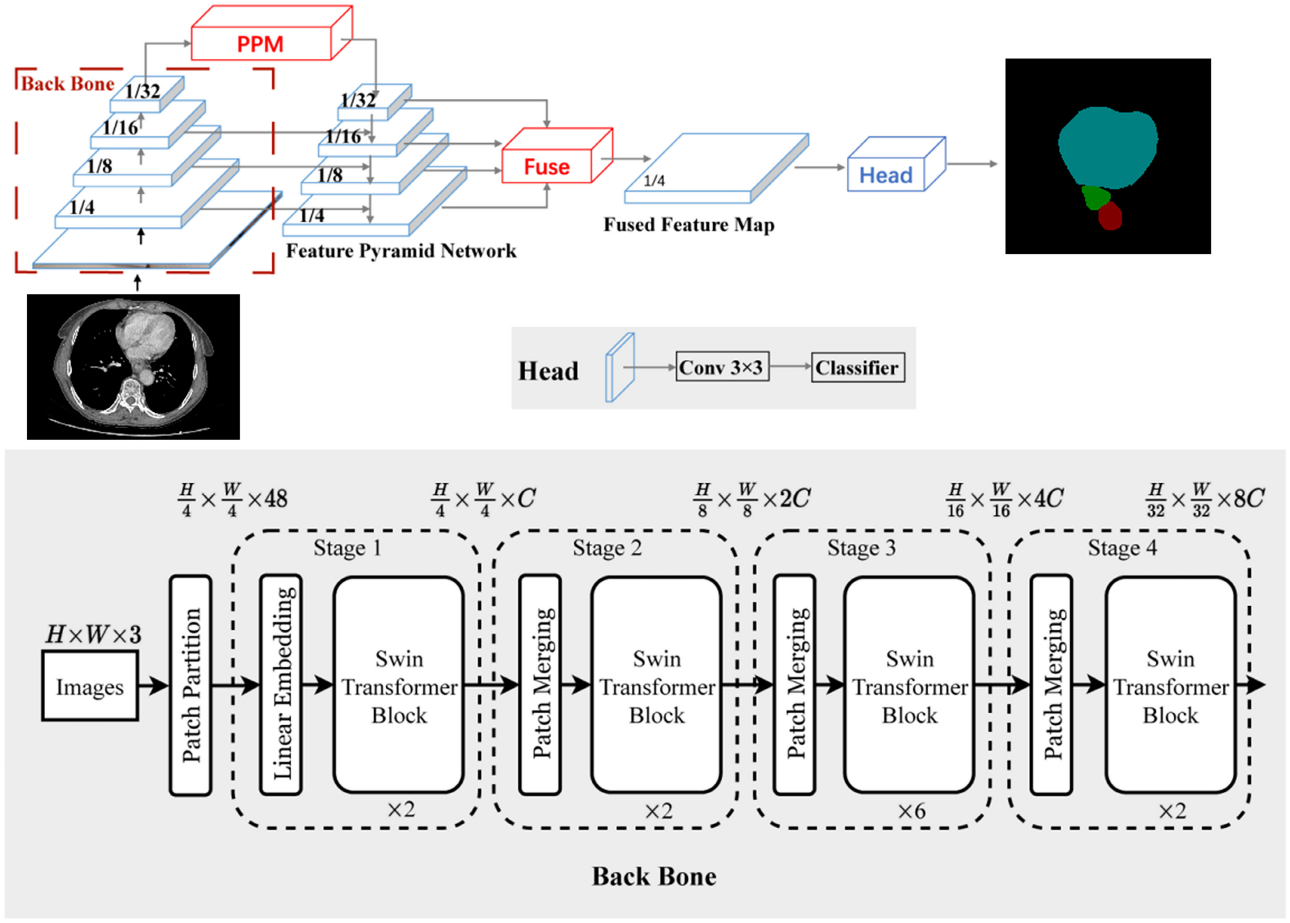
UperNet Swin network structure. First, the preprocessed original CTA image and the mask containing the ground truth are input, and feature extraction is carried out through the Back Bone module. The global information is fully utilized through PPM to enter the FPN for feature fusion, and the fused image features are obtained through convolution and classifier to obtain the final prediction map.

The preprocessed image was input into the UperNet Swin network for modeling. Using the UperNet Swin network with UperNet as the basic framework and the Swin Transformer as the backbone network has significantly enhanced its feature extraction ability. Our study applies this network to the segmentation of EC and its adjacent structures for the first time, and the specific structure is shown in Figure 2.

UperNet was based on the feature pyramid network (FPN).^18^ FPN is a general‐purpose feature extractor with simple performance. The feature map is only upsampled by bilinear interpolation rather than time‐consuming deconvolution, and the top‐down path is fused with the bottom‐up path into 1 × 1 convolutional layers, followed by element‐by‐element summation, without any complex refinement module. Its simplicity is what achieves its efficiency.^26^ The function of the PPM (Pyramid pooling module) structure is to make full use of global information to prevent the model from overlearning large‐scale goals such as the pericardium while ignoring small‐scale goals such as tumors. Image features enter the FPN after passing through the PPM, and in the lower process, the features communicate with those generated in the upper process. Then, the features generated in each layer of the lower process are fused, and the fused image features are passed through convolution and classifier to obtain the final prediction map.^26^, ^27^

The Swin Transformer is a layered vision deformer that uses a shifted window to serve as a general‐purpose backbone for computer vision. Similar to the hierarchical feature maps in convolutional neural networks, such as the feature map size having 4 times, 8 times and 16 times the downsampling of the image, such a backbone helps to build object detection, instance segmentation and other tasks on this basis.^19^ During the training process, we use data augmentation strategies such as image rotation, scaling, brightness, contrast, gamma, and mirroring to improve the segmentation performance of the model.

All experiments are based on the Python language, PyTorch deep learning framework, and the model was built and trained. During the model training phase, data augmentation was performed using random rotation, random horizontal flipping, random brightness, and contrast transformations. Cross entropy loss was used during training, the optimizer selected Adam, the initial learning rate was 0.001, and the GPU for training the model used Quadro RTX 5000.

### 2.4 Statistical Analysis

We used one‐way ANOVA to compare continuous variables and chi‐square tests or Fisher's exact tests to compare categorical variables.^28^, ^29^ Continuous variables are represented as the mean ± standard deviation, and categorical variables are represented as numbers and percentages. A *p*‐value <0.05 indicated statistical significance. All statistical analyses in this study were performed using SPSS 25.0. We could also calculate the predicted volume of EC based on the EC segmentation results and compared the predicted volume with the volume recorded in the ground truth.

### 2.5 Model evaluation indicators

We use metrics such as the Dice similarity coefficient (DSC), sensitivity and positive predictive value (PPV) to evaluate the predictive performance of the model.^30^ DSC is a statistic used to evaluate the similarity of two samples, essentially measuring the overlapping part of two samples and evaluating the performance of the spatial overlap accuracy of the manual segmentation and automatic segmentation methods.^31^ When the two masks of the test set and the training set coincide, the values of DSC, sensitivity and PPV are 1, and the segmentation results are best. The model segmentation evaluation index is calculated as follows:

$$\mathrm{DSC}=\frac{2\left|P\cap T\right|}{\left|P\right|+\left|T\right|}$$

$$\text{Sensitivity}=\frac{\left|P\cap T\right|}{\left|T\right|}$$

$$\mathrm{PPV}=\frac{\left|P\cap T\right|}{\left|P\right|}$$

where GT denotes the manually annotated ground truth, and Pred denotes the model segmented prediction. |Pred| and |GT| represent the areas of Pred and GT, respectively.^30^ |Pred∩GT| denotes the spatial overlap region between Pred and GT.

## 3 RESULTS

### 3.1 Statistical analysis of the dataset

A total of 182 patients were included, and we randomly divided the data into a training set and a test set at a ratio of 8:2. There were no significant differences between the training and test sets in the characteristics of age, sex, T stage, N stage, tumor location, clinical stage, neurovascular invasion, and degree of pathological differentiation (*p* > 0.05). The patient characteristics are summarized in Table 1, and these characteristics were well balanced between the two cohorts.

### 3.2 Comparison of the performance of different network models for segmenting EC

We used 4 models for training independently under the same experimental protocol.

**FIGURE 3:**
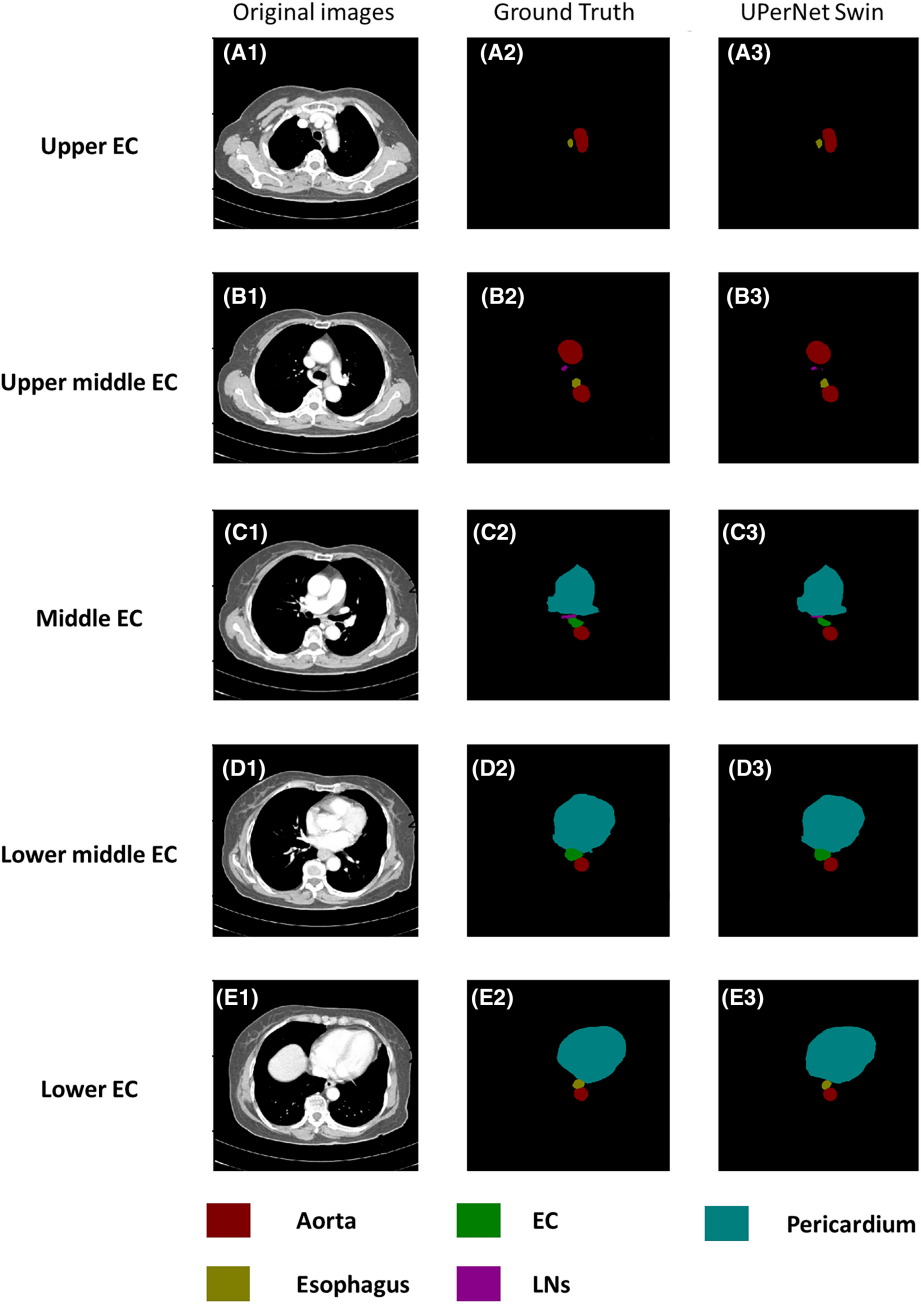
Comparison of EC predictions in different parts of the upernet switch network model. A1–A3 are the original image, gold standard and model prediction map of the upper EC. B1–B3 are the original image, gold standard and model prediction chart of middle and upper EC. C1–C3 is the original image, gold standard and model prediction map of mid‐stage EC. D1–D3 is the original image, gold standard and model prediction map of middle and lower EC. E1–E3 are the original image, gold standard and model prediction map of the lower EC.

**FIGURE 4:**
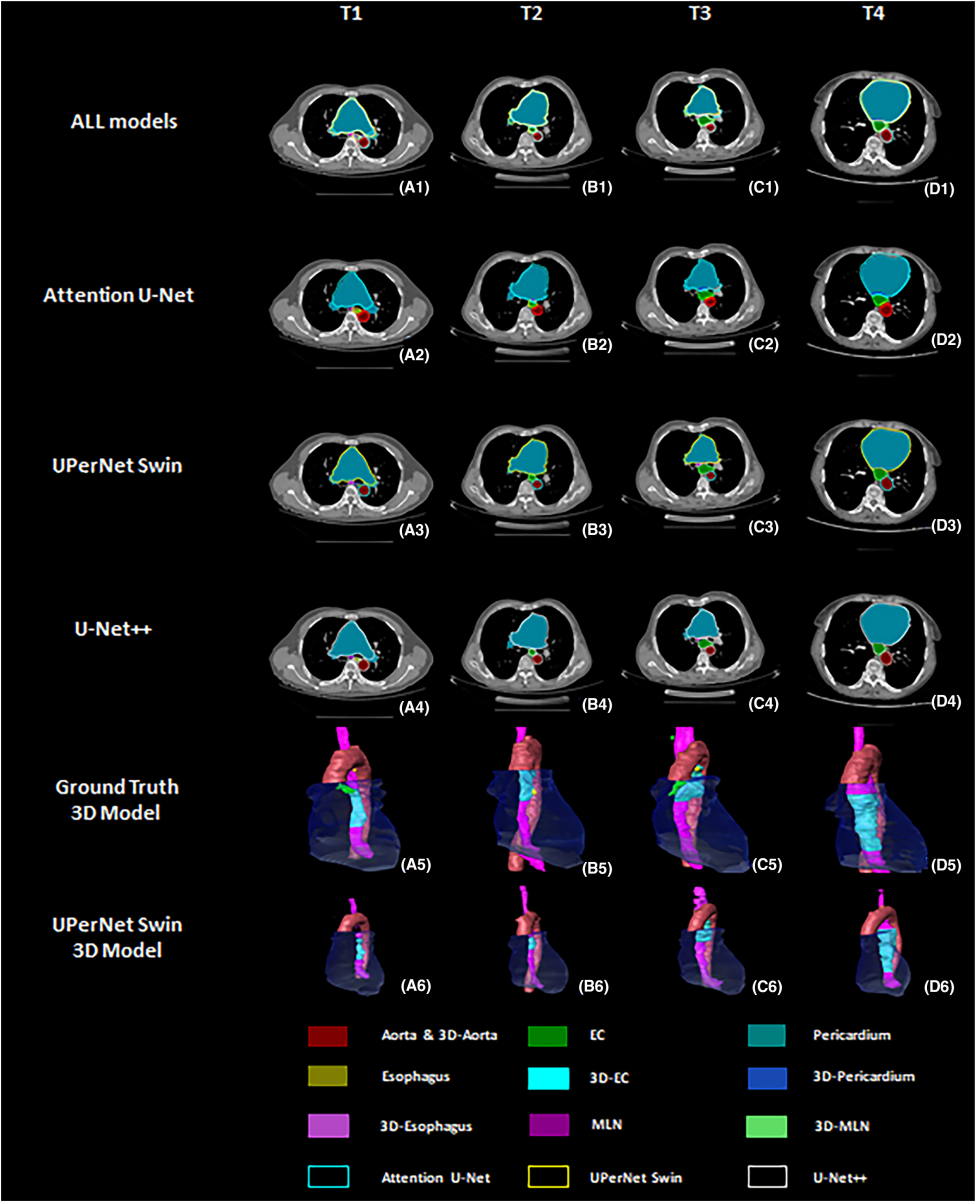
2D segmentation and 3D reconstruction results of EC with different T staging on four models. A1–D1 are the segmentation results of the gold standard and all models superimposed on one piece. A2–D2 is the segmentation result comparison of the gold standard and attention U‐Net model. A3–D3 is the segmentation result comparison of the gold standard and UperNet Swin model. A4–D4 is the segmentation result comparison of the gold standard and Unet++ model. A5–D5 are the 3D reconstruction models of the gold standard; A6–D6 are the 3D reconstruction models of the segmentation result of the UperNet Swin model.

Table 2 reports the results of the comparison. The UperNet Swin network achieved the best performance with a DSC of 0.782 for EC and 0.8033 and 0.7618 for sensitivity and PPV, respectively, which were superior to the values of the Attention U‐Net, Unet++, and Unet models, with DSC values of 0.6479, 0.6761, and 0.6666, respectively. (Figures 3 and 4).

**TABLE 2:** Quantitative segmentation results of different network models for each structure on the test set.

	Attention U‐Net	UperNet Swin	Unet++	Unet
EC				
DSC	0.6479	**0.7820**	0.6761	0.6666
Sensitivity	0.6452	**0.8033**	0.6896	0.7641
PPV	0.6506	**0.7618**	0.6631	0.5912
Esophagus				
DSC	**0.7446**	0.7298	0.7381	0.7090
Sensitivity	**0.7149**	0.6815	0.6905	0.6308
PPV	0.7768	**0.8100**	0.7928	0.8093
Pericardium				
DSC	0.9636	**0.9664**	0.9641	0.9635
Sensitivity	0.9662	**0.9707**	0.9641	0.9630
PPV	0.9610	0.9620	**0.9640**	0.9639
Aorta				
DSC	0.9439	**0.9496**	0.9454	0.9465
Sensitivity	0.9441	0.9525	0.9469	**0.9532**
PPV	0.9438	0.9467	0.9438	**0.9488**
Lymph node				
DSC	**0.5359**	0.5091	0.3153	0.5141
Sensitivity	**0.5965**	0.4256	0.2516	0.4097
PPV	0.4866	0.6334	0.4221	**0.6901**

Abbreviations: DSC, Dice Similarity Coefficient; EC, esophageal cancer; PPV, positive predictive value.

*Note:* The bolded values indicate the highest values of DSC, sensitivity and PPV for different structures of esophageal cancer and its adjacent structures in different networks.

**FIGURE 5:**
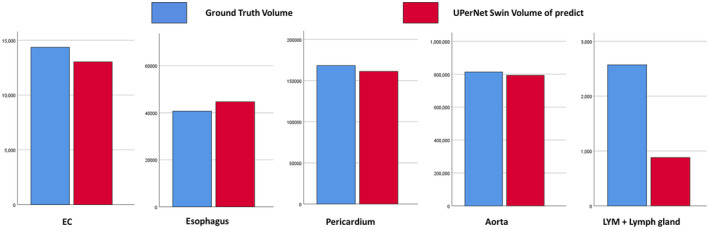
Volume comparison of different structures in the UperNet Swin model with the gold standard.

**FIGURE 6:**
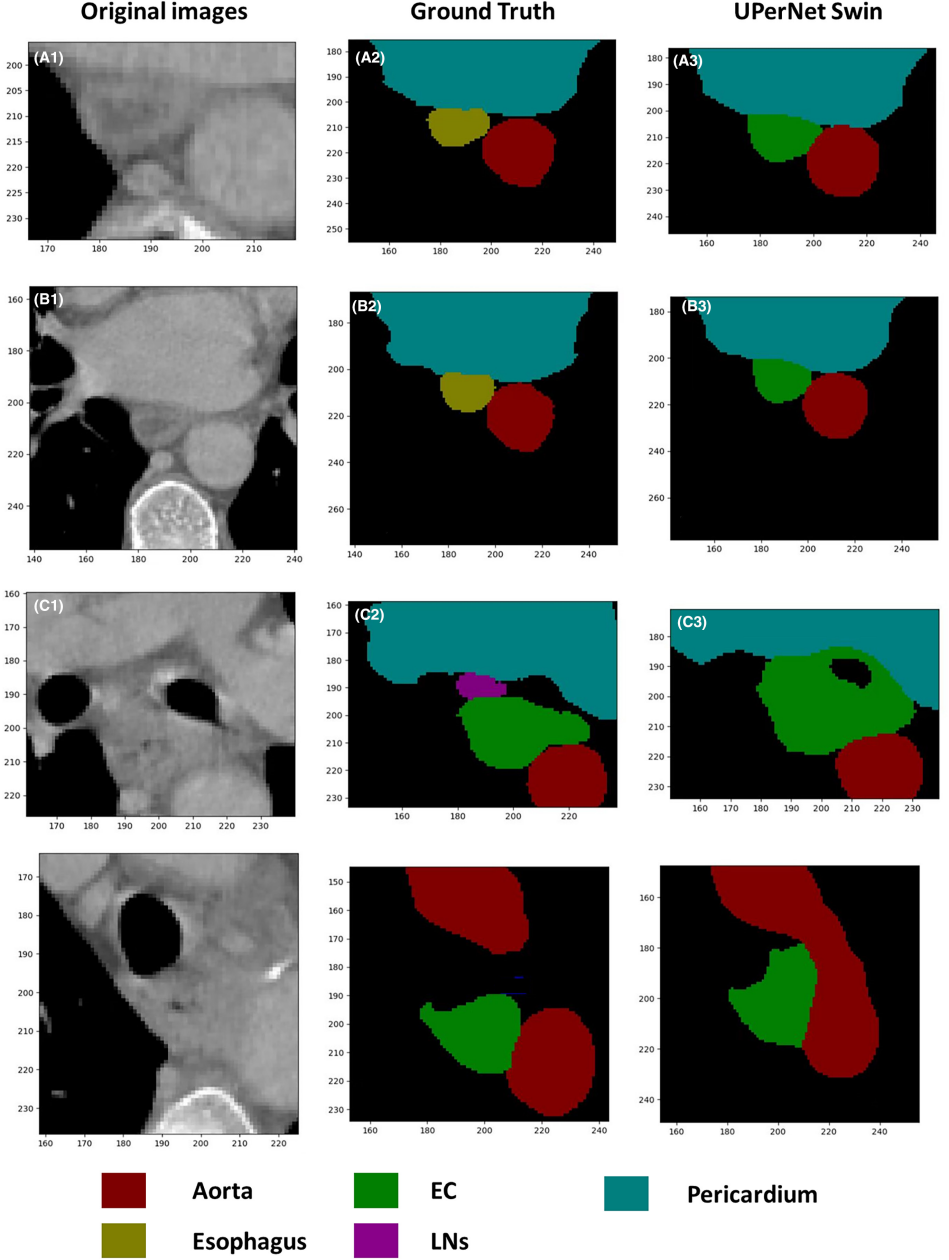
Recognition of the tumor boundary by the UperNet Swin model and manual segmentation. A1–A3 and B1–B3 are the mid‐thoracic esophageal planes, and the gold standard is the normal esophagus, which is automatically segmented as tumor lesions by the model; C1–C3 and D1–D3 are the tracheal ridge planes, and the gold standard is the tumor and enlarged lymph nodes, which are jointly recognized as tumor by the model. The location of lymph nodes was not clearly distinguished, resulting in excessive tumor volume.

The highest DSC value of EC was 0.7820 on the UperNet Swin network model and 0.6479, 0.6761, and 0.6666 on the Attention U‐Net, UNet++, and UNet, respectively. The esophagus had DSC values of 0.7446, 0.7298, 0.7381, and 0.7090 on all four models. The DSC values of the pericardium were above 0.96 on all four models, with the lowest value of 0.9635 for Attention U‐Net and the highest value of 0.9664 for UperNet Swin. The highest DSC value for the aorta was 0.9496 for the UperNet Swin, and the lowest value was 0.9439 for the Attention U‐Net. The DSC values of MLN were 0.5359, 0.5091, 0.3153, and 0.5141, with the UperNet Swin model having the highest values. (Table 2; Figures 5 and 6).

For the sensitivity of segmentation performance, the sensitivity of the four models for EC segmentation was 0.6452, 0.8033, 0.6896, and 0.7641, with the highest value for the UperNet Swin. The model with the highest sensitivity for pericardium segmentation was the UperNet Swin, with a value of 0.9707. The model with the highest sensitivity for MLN and esophagus segmentation was the Attention U‐Net, with values of 0.5965 and 0.7149, respectively. The UNet had the highest sensitivity for aorta segmentation, with a value of 0.9532.

Regarding the values of PPV, the values of EC were 0.6506, 0.7618, 0.6631, and 0.5912, respectively, with the UperNet Swin performing the best. The esophagus had the highest PPV on the UperNet Swin with a value of 0.8100; aorta and LNs had the Unet with the highest PPV with values of 0.9488 and 0.6901, respectively. The pericardium had a PPV of 0.9640, and the attention U‐Net had the highest values.

Among the four network models, the overall segmentation results of the UperNet Swin network were better than those of the other models, showing better DSC values of 0.6164, 0.7842, 0.8190 and 0.7259 in T1‐T4 staging, respectively. The UNet was better than the other three models in terms of sensitivity to EC (T1‐T3), and the UperNet Swin performed better regarding the sensitivity of T4. The PPV values were also higher than those of the other models on both T1 and T3, which were not as good as those of the other models; the attention U‐Net showed the best performance on the PPV of T4 (Table 3).

**TABLE 3:** Quantitative EC segmentation results of different network models on different T‐stages.

	Attention U‐Net	UperNet Swin	Unet++	Unet
T1				
DSC	0.5220	**0.6164**	0.3793	0.5663
Sensitivity	0.3087	0.3847	0.3524	**0.4120**
PPV	0.6191	**0.7404**	0.5108	0.4810
T2				
DSC	0.6102	**0.7842**	0.6787	0.7563
Sensitivity	0.4977	0.6079	0.5633	**0.7189**
PPV	**0.8670**	0.8157	0.8535	0.7977
T3				
DSC	0.6685	**0.8190**	0.6856	0.6670
Sensitivity	0.7301	0.7747	0.7472	**0.8230**
PPV	0.6165	**0.7913**	0.6334	0.5607
T4				
DSC	0.6730	**0.7259**	0.7129	0.7084
Sensitivity	0.5985	**0.7185**	0.6801	0.6806
PPV	**0.7687**	0.7361	0.7490	0.7385

*Note:* The bolded values indicate the highest values of DSC, sensitivity and PPV in different networks for different T‐stages of esophageal cancer.

Abbreviations: DSC, Dice Similarity Coefficient; EC, esophageal cancer; PPV, positive predictive value.

### 3.3 Comparison of manually segmented and automatically segmented volumes

We compared the automatically segmented volumes of the model with the manually segmented volumes from different T stages and different structures.

The manually segmented volume values for T1‐T4 stages were 7.34 ± 1.78, 17.03 ± 9.70, 23.16 ± 13.88 and 35.41 ± 37.56 cm^3^, and the automatically segmented volume values for the UperNet Swin were 7.90 ± 4.74, 12.59 ± 10.81, 27.74 ± 32.96 and 37.58 ± 16.06 cm^3^, which were not significantly different (*p* > 0.05). (Table 4).

**TABLE 4:** Volume comparison between automatic and manual segmentation of the UperNet Swin model in different T‐stages.

T‐Stage	Volume of ground truth (cm^3^)	Volume of predict(cm^3^)	*F*	*p*‐value^a^
		UperNet Swin		
T1	7.34 ± 1.78	7.90 ± 4.74	2.323	0.153
T2	17.03 ± 9.70	12.59 ± 10.81	1.402	0.247
T3	23.16 ± 13.88	27.74 ± 32.96	1.438	0.242
T4	35.41 ± 37.56	37.58 ± 16.06	1.691	0.226

*Note*: Date is mean ± SD.

^a^ *p* < 0.05 is considered statistically significant.

The GT volume of EC was 18.56 ± 2.16 cm^3^, and the predicted volume of the UperNet Swin was 19.31.88 ± 25.49 cm^3^, with *p* = 0.305 (*p* > 0.05), indicating no significant difference between them. The GT volumes for the esophagus, pericardium, and aorta were 40.85 ± 8.36, 884.27 ± 398.75 and 166.65 ± 293.88 cm^3^, respectively, and the predicted values were 44.79 ± 11.93, 781.46 ± 157.19, 159.24 ± 31.14 cm^3^, respectively; all of them had p values greater than 0.05, indicating that none of the p values were statistically significant. The predicted GT and MLN volumes were 4.53 ± 8.95 and 0.89 ± 0.52 cm^3^, respectively, with *p* = 0.002, and the difference between the two values was statistically significant. (Table 5).

**TABLE 5:** Volume comparison of UperNet Swin model with manual segmentation on segmentation of different structures.

Type	Volume of ground truth (cm^3^)	Volume of predict(cm^3^)	*F*	*p*‐value^a^
		Upernet Swin		
EC	18.56 ± 2.16	19.31 ± 25.49	1.073	0.305
Esophagus	40.85 ± 8.36	44.79 ± 11.93	3.538	0.065
Pericardium	884.27 ± 398.75	781.46 ± 157.19	1.099	0.299
Aorta	166.65 ± 29.39	159.24 ± 31.14	0.007	0.932
MLN	4.53 ± 8.95	0.89 ± 0.52	10.076	**0.002**

*Note*: Date is mean ± SD. bolded values indicate differences between the volumes of the Upernet Swin network and the hand‐segmented MLN. It shows that the network is not very accurate in segmenting small structures, and the difference with manual segmentation is large, which is the main direction of our optimization of the model afterwards.

Abbreviations: EC, esophageal cancer; MLN, Metastatic Lymph Nodes.

^a^ *p* < 0.05 is considered statistically significant.

### 3.4 Segmentation Time Comparison

The trained model takes approximately 18 s to finish EC automatic segmentation, while the manual segmentation time is 1135.32 ± 435.78 s. The UNet++ takes the least time to segment each patient, approximately 5.03 s, the UperNet Swin takes the longest time, 17.9 s, and the Attention U‐Net and UNet are in the middle, taking 8.23 and 10.87 s, respectively. (Table S1).

## 4 DISCUSSION

### 4.1 Advantages and significance of our deep learning model

In this study, we propose a deep learning model, UperNet Swin, for automated segmentation and volume analysis of different T stages in EC patients and adjacent structures around EC. Our model achieves good performance in segmenting and measuring the volume of EC, approaching and reaching the radiologist level. In addition, our model is computationally efficient, taking only 18 s to segment and measure the volume of a patient's EC and its surrounding adjacent structures, while it takes up to 1 h for a clinician or radiologist to accurately segment the volume of each patient, greatly saving physician workload and medical resources.

The UperNet Swin has the highest EC DSC values and performs best in the task of segmenting tumors. This is because the UperNet Swin network uses the Swin Transformer structure in each feature extraction module, and the Swin Transformer module has a strong feature extraction ability, which to a certain extent solves the problems of less feature extraction and poor image quality of the UperNet and improves the image segmentation performance.^18^, ^19^ We also compared the DSC values of the four models in different structures, including the EC, esophagus, pericardium, aorta, and lymph gland, and found that the pericardium and aorta had the highest DSC, the lymph gland had the lowest DSC value, and the EC and esophagus DSC values were in the middle because the pericardium and aorta structures were relatively large. The structure outline was clear, and the boundary with the surrounding tissue was obvious and was easy to identify in the CTA images. Therefore, its DSC value is relatively higher. Compared with the pericardium and aorta, lymph nodes look very small, with an average of only 4.53 cm^3^, and their locations are not fixed and are not easy to identify, so the DSC value of metastatic lymph nodes is low.

### 4.2 Comparison with previous studies

Previous studies have shown that deep learning can improve the consistency and save time in the depiction of tumor volume profiles and surrounding organs at risk in cerebral hemorrhage,^32^ nasopharyngeal carcinoma,^33^ cervical cancer,^34^, ^35^ breast cancer,^36^, ^37^ rectal cancer,^38^, ^39^ pulmonary blood vessels,^40^ and lung cancer.^41^, ^42^ Therefore, one of our main goals was to accurately segment and quantify tumor volume size in EC patients, and the UperNet‐Swin performs well in segmenting and measuring EC volume, which is consistent with previous deep learning studies on EC.^21^, ^22^, ^43^, ^44^, ^45^ Our study cannot only accurately quantitatively assess EC but also accurately helps with EC T‐stage diagnosis, prognostic assessment and treatment decision‐making.

While several previous studies have used machine learning methods to segment tumors in EC patients, they did not segment the MLN of EC and the adjacent structures surrounding the tumor. To automatically identify the GTV outline of 215 EC patients, Jin L et al.^21^ used three deep learning models: 2DU‐Net, 3D V‐Net, and VUMix‐Net. Their study indicated that the DSC of the VUMix‐Net mixed model was marginally higher than that of the single network model, with a DSC value of 0.68. However, in our study, the automatic segmentation of EC using the UperNet Swin network had a DSC value of 0.782, which was higher than their model. We also performed volume analysis on EC and not only EC positioning diagnosis, but we also calculated the EC volume based on the results of automatic segmentation, evaluated EC's T stage, made up for the shortcomings of their research, and more comprehensively located and diagnosed EC. Zhang P. et al.^22^ used a deep learning system (DLS) to detect EC on barium esophagrams and dichotomous images with an accuracy of 0.837. Gong EJ et al.^44^ used endoscopic images to establish a deep learning model for diagnosing EC, esophageal dysplasia and inflammatory esophagus, and the accuracy of the model for diagnosing EC was 0.78. The results of our model agree with the results of Zhang P. et al. and Gong EJ, et al., but they only used one deep learning model to diagnose EC and did not use more models for training. Therefore, our advantage is that we selected the optimal model to segment EC, which improves the efficiency of identification and segmentation.

Overall, the UperNet Swin performed best overall in the segmentation task for EC and its adjacent structures. The UNet++ and UNet are better for segmentation of large structures such as the esophagus, pericardium and aorta, while the attention U‐Net gives better results for the segmentation of small structures such as MLN. Wu L. et al.^46^ used radiomics and CT images for the prediction of EC of the MLN. In contrast to their study technique, we employed a deep learning network for MLN segmentation with a DSC value of 0.5359, which was less successful. Because the MLN was tiny and not easy to identify and locate from connective tissue, autosegmentation was difficult. The fact that the DSC value of the T2‐T3 stage is higher than that of the T1 and T4 stage may be due to the larger sample sizes of the T2 and T3 groups. Tumors in T1 are relatively smaller and cannot be autoidentified and autosegmented easily, and tumors in T4 have irregular 2D and 3D morphology, which cannot be autosegmented accurately. The UperNet Swin takes the longest time, probably because it extracts more tumor features, and it takes more time to integrate these features to improve segmentation results and obtain better DSC values.

## 5 LIMITATIONS

First, although our data came from two different centers, the relatively small amount of data from Shanxi Cancer Hospital resulted in less balanced data, so in the future, we will continue to increase the amount of data. Second, the DSC values of the model are still not very good, especially on the upper and lower boundaries of ECs, and when the two tissues of ECs and LNs are close to each other, the network recognizes them poorly. Third, the doctors defined the top and lower limits of EC by combining the findings from CTA, gastroscopy, barium esophageal meal, and even PET‐CT. The model's inaccuracy in identifying the upper and bottom bounds of EC is because it could only learn to delineate them from CTA images.

## 6 CONCLUSION

In general, we use the UperNet Swin network to create an intelligent segmentation model for EC, which achieves good results in segmenting EC and its surrounding adjacent structures, and it performs better than the Attention U‐Net, UNet++, UNet and VUMix‐Net. The autosegmentation accuracy is close to manual segmentation, which is helpful for accurate T‐stage diagnosis of EC, which can reduce clinician workload, improve work efficiency, and save medical resources.

## AUTHOR CONTRIBUTIONS

**Runyuan Wang:** Data curation (equal); formal analysis (equal); resources (equal); software (equal); visualization (equal); writing – original draft (equal). **Xingcai Chen:** Data curation (equal); software (equal). **Xiaoqin Zhang:** Formal analysis (equal); methodology (equal). **Ping He:** Resources (equal). **Jinfeng Ma:** Resources (equal). **Huilin Cui:** Funding acquisition (equal); resources (equal). **Ximei Cao:** Resources (equal). **Yongjian Nian:** Software (equal). **Ximing Xu:** Funding acquisition (equal). **Wei Wu:** Funding acquisition (equal); methodology (equal); resources (equal). **Yi Wu:** Funding acquisition (equal); methodology (equal); project administration (equal).

## FUNDING INFORMATION

This work was supported by University Funded Science and Technology Innovation Capacity Improvement Project (No. 2019XYY14), Science‐Health Joint Medical Scientific Research Project of Chongqing (No. 2022ZDXM018), the National Natural Science Foundation of China under Grant (31971113), the Chongqing Science and Technology Talent Project (No. CQYC201905037), Basic Research (Free Exploration) Project of Shanxi Province (2021011079‐2) and the Health Project of National Clinical Research Center for Child Health and Disorders(Children's Hospital of Chongqing Medical University, Chongqing, China) (Grant number:NCRCCHD‐2022‐HP‐01).

## Supporting information


Data S1.

## Data Availability

Data available on request from the authors: The data that support the findings of this study are available from the corresponding author upon reasonable request.
