# Supplementary material for: Automatic segmentation of esophageal cancer, metastatic lymph nodes and their adjacent structures in CTA images based on the UperNet Swin network
Source: Cancer Med. 2024 Sep 20;13(18):e70188. doi: 10.1002/cam4.70188 (PMC11413407; doi:10.1002/cam4.70188)
Supplement: Supplementary file 1 — Data S1. [file CAM4-13-e70188-s001.docx]

The trained model takes approximately 18s to provide EC automatic segmentation and volume analysis for each patient, while the average manual segmentation time is 1135.32±435.78s. The Unet++ model had the least time to segment each patient, about 5.03s, the UperNet Swin model with the longest time at 17.9s, and Attention U-Net and Unet in the middle, which required 8.23s and 10.87s. (Supplemental table 1)

Supplemental table 1. Time taken by different models compared to manual sketching time

|  | Attention U-Net | UperNet Swin | Unet++ | Unet | Manual |
| --- | --- | --- | --- | --- | --- |
| Total(s) | 8.23 | 17.9 | 5.03 | 10.87 | 1135.32±435.78 |

Date is mean±SD.


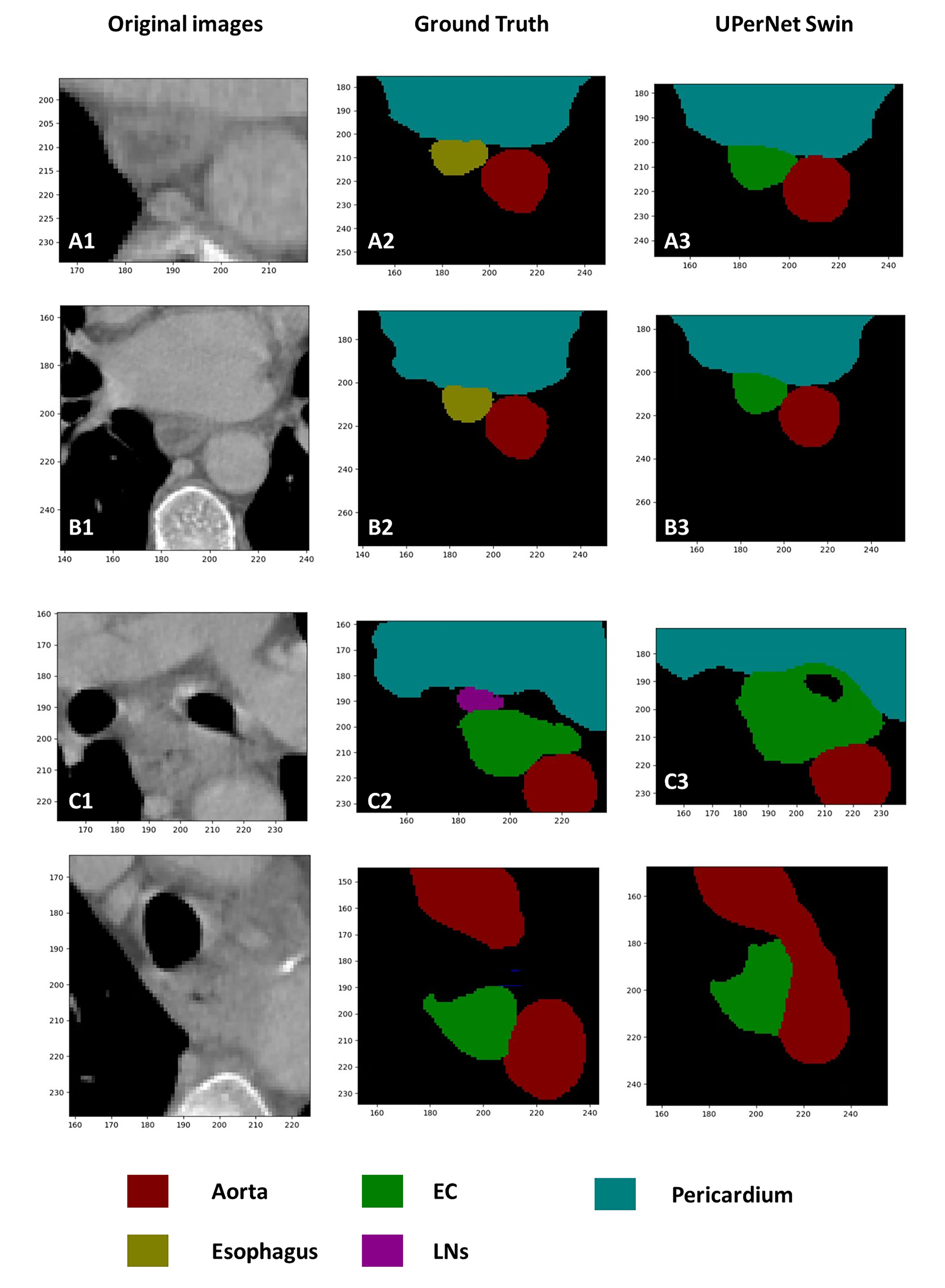


Figure 6. Recognition of the tumor boundary by the UperNet Swin model and manual segmentation

A1-A3 and B1-B3 are the mid-thoracic esophageal planes, and the gold standard is the normal esophagus, which is automatically segmented as tumor lesions by the model; C1-C3 and D1-D3 are the tracheal ridge planes, and the gold standard is the tumor and enlarged lymph nodes, which are jointly recognized as tumor by the model. The location of lymph nodes was not clearly distinguished, resulting in excessive tumor volume.
